# Robotic- versus laparoscopic-assisted distal gastrectomy with D2 lymphadenectomy for advanced gastric cancer based on propensity score matching: short-term outcomes at a high-capacity center

**DOI:** 10.1038/s41598-020-63616-1

**Published:** 2020-04-16

**Authors:** Shan-ping Ye, Jun Shi, Dong-ning Liu, Qun-guang Jiang, Xiong Lei, Bo Tang, Peng-hui He, Wei-quan Zhu, He-chun Tang, Tai-yuan Li

**Affiliations:** 1 Department of General Surgery, First Affiliated Hospital of Nanchang University, No. 17 Yongwaizheng Street, Nanchang, Jiangxi Province 330006 China; 2 Department of Graduate School, Medical College of Nanchang University, No. 461 Bayi Avenue, Nanchang, Jiangxi Province 330006 China

**Keywords:** Gastric cancer, Gastroenterology

## Abstract

Reports in the field of robotic surgery for gastric cancer are increasing. However, studies only on patients with advanced gastric cancer (AGC) are lacking. This retrospective study was to compare the short-term outcomes of robotic-assisted distal gastrectomy (RADG) and laparoscopic-assisted distal gastrectomy (LADG) with D2 lymphadenectomy for AGC. From December 2014 to November 2019, 683 consecutive patients with AGC underwent mini-invasive assisted distal gastrectomy. Propensity-score matching (PSM) analysis was conducted to reduce patient selection bias. Short-term outcomes were compared between the two groups. The clinical features were well matched in the PSM cohort. Compared with the LADG group, the RADG group was associated with less operative blood loss, a lower rate of postoperative blood transfusion, less volume of abdominal drainage, less time to remove abdominal drainage tube, retrieved more lymph node, and lower rates of surgical complications and pancreatic fistula (*P* <0.05). However, the time to recovery bowel function, the length of postoperative stay, the rates of other subgroups of complications and unplanned readmission were similar between the two groups (*P* > 0.05). This study suggests that RADG is a safe and feasible technique with better short-term outcomes than LADG for AGC.

## Introduction

Gastric cancer continues to be a major public health problem worldwide, especially in developing countries, such as China^1,2^. Every year, approximately 680,000 new patients are diagnosed with gastric cancer in China; among them, more than 80% of these patients have advanced-stage disease^1,3^. For patients with advanced gastric cancer (AGC), multidisciplinary comprehensive treatment is usually required, and gastrectomy with lymph node dissection is currently considered to be the only curable treatment^4^.

In the past few decades, with the development of science and technology, much advanced surgical equipment has been invented, particularly in the field of mini-invasive surgery (MIS). Since laparoscopy-assisted Billroth I gastrectomy was first reported in 1994, several randomized controlled trials (RCTs) have demonstrated that laparoscopic gastrectomy (LG) for AGC is a safe and feasible technique with better short-term outcomes than and similar oncological outcomes to open gastrectomy^5–9^. Although LG has obtained greater acceptance among abdominal surgeons, because of the limitations of laparoscopic instruments, it is difficult to perform precisely, as is the case with D2 lymphadenectomy^10,11^.

Robotic surgery systems, as another MIS method, were invented to overcome the drawbacks of laparoscopy and are becoming increasingly accepted by abdominal surgeons. Since Hashizume *et al*. first reported the use of robotic surgery for gastric cancer in 2002, a number of studies have shown the safety and advantages of robotic gastrectomy^12–14^. However, most of these studies have analyzed patients who were at a relatively early stage of disease. In fact, reports only on RADG for AGC are lacking. Therefore, to evaluate the safety and advantages of RADG for AGC, we designed this retrospective cohort study with a large sample size to compare the short-term outcomes and postoperative complications of RADG and LADG with those of D2 lymph node dissection for AGC in a high-capacity center in China.

## Methods

### Patients

The Da Vinci^R^ Robot Surgical System was introduced into our center (Department of General Surgery, The First Affiliated Hospital of Nanchang University) in December 2014. From then on, patients who had minimally invasive distal gastrectomy planned could choose their preferred surgical approach after being informed by the surgeon about the advantages and disadvantages of RADG and LADG. The current retrospective cohort study was approved by the institutional review board of our hospital and complies with the Helsinki Declaration. All patients signed written informed consent voluntarily before the operation.

**Figure 1:**
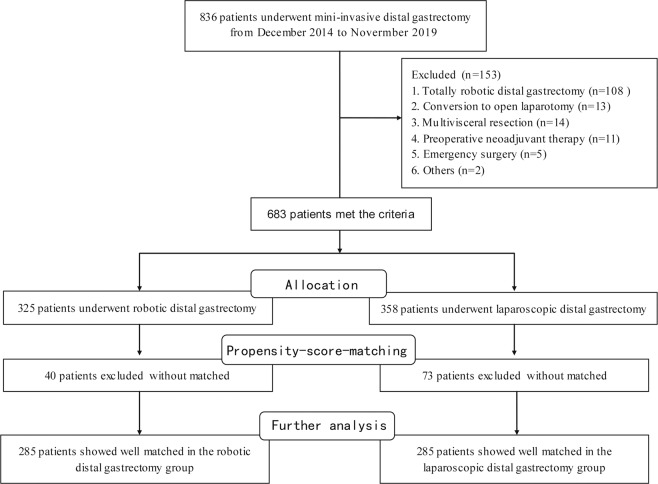
Flow chart of patient selection.

From December 2014 to November 2019, 836 patients with AGC (T2-4aN0-3M0 according to the 8^th^ edition of the American Joint Committee on Cancer criteria) underwent mini-invasive distal gastrectomy at our center. The exclusion criteria were as follows: (1) conversion to open laparotomy; (2) multivisceral resection; (3) totally robotic surgery or totally laparoscopic surgery; (4) preoperative neoadjuvant therapy; (5) emergency surgery; (6) robotic or laparoscopic equipment failure during operation; and (7) incomplete clinical records of patients. After exclusion, data from 683 patients were retrospectively collected for further analysis, including date from 325 patients in the RADG group and 358 patients in the LADG group. To reduce the effect of patient selection bias between the two surgical methods, we conducted PSM based on a linear model with a caliper value of 0.01 (one-to-one nearest neighbor matching)^15,16^. Covariate analysis of the linear model included all the clinical characteristics shown in Table 1. Ultimately, 570 patients were enrolled in the current study, with 285 patients in each group, and a flow chart of the patient selection scheme is shown in Fig. 1.

**Table 1:** Patient’s clinicopathological characteristics of RADG and LADG group for AGC.

Characteristics	RADG (n = 285)	LADG (n = 285)	*P* value
Gender, n (%)			0.791^a^
Male	189 (66.3)	186 (65.3)	
Female	96 (33.7)	99 (34.7)	
Age, years	57.1 ± 8.3 (41.0–80.0)	57.0 ± 8.6 (41.0–80.0)	0.851^b^
Body mass index, kg/m^2^	24.4 ± 2.3 (19.9–29.0)	24.5 ± 2.2 (19.7–29.6)	0.623^c^
Type of reconstruction, n (%)			0.280^a^
B-I	72 (25.3)	82 (28.8)	
B-II	184 (64.6)	166 (58.2)	
Roux-en-Y	29 (10.2)	37 (13.0)	
T stage, n (%)			0.529^a^
2	45 (15.8)	54 (18.9)	
3	95 (33.3)	97 (34.0)	
4a	145 (50.9)	134 (47.0)	
N stage, n (%)			0.105^a^
0	82 (28.8)	61 (21.4)	
1	101 (35.4)	110 (38.6)	
2	70 (24.6)	88 (30.9)	
3	32 (11.2)	26 (9.1)	
pTNM, n (%)			0.417^a^
I-B	7 (2.5)	4 (1.4)	
II-A	64 (22.5)	57 (20.0)	
II-B	85 (29.8)	89 (31.2)	
III-A	49 (17.2)	65 (22.8)	
III-B	53 (18.6)	51 (17.9)	
III-C	27 (9.5)	19 (6.7)	
ASA classification, n (%)			0.715^a^
1	168 (58.9)	161 (56.5)	
2	102 (35.8)	111 (38.9)	
3	15 (5.3)	13 (4.6)	
Diameter of neoplasm, mm	48.4 ± 13.1 (20.0–81.0)	48.6 ± 13.1 (20.0–81.0)	0.987^c^

*AGC*: Advanced gastric cancer; *ASA*: American Society of Anesthesiologists; *LADG*: laparoscopic assisted distal gastrectomy; *RADG*: robotic assisted distal gastrectomy; *TNM*: tumor node metastasis staging.

^a^Pearson’s chi-squared test.

^b^Student’s t test.

^c^Mann–Whitney U test.

All of the patients were diagnosed, staged, and evaluated using preoperative electronic gastroscopy and biopsy and chest and abdominopelvic enhanced computed tomography. The clinical baseline characteristics (Table 1), short-term outcomes (Table 2), and postoperative complications (Table 3) of the patients were extracted from the database maintained at our center and compared between the RADG and LADG groups. Two team (each with three authors) independently collected above data, all disputes were resolved by negotiations. The total operative time was defined as the time from the beginning of sterilization of the area of operation to completion of the skin suture, the setup time was defined as the time from the beginning of sterilization of the area of operation to the completion of the Trocar placement, the time from the beginning of peritoneal exploration to the withdrawal of the device was defined as the total time needed for the mini-invasive surgical resection, and the laparotomy operation time was defined as the time from the beginning of withdrawal of the device to the completion of the skin suture. Any deviation from the normal postoperative procedure was considered as a postoperative complication^17^. A clinically relevant postoperative pancreatic fistula (POPF) was defined as abdominal drainage fluid with amylase activity three times higher than the upper limit of normal serum value^18^. And, its grading was referred to the update consensus of International Study Group on Pancreatic Surgery (ISGPS)^18^. Patients with complications refer to the number of patients who had complications. The number of overall complications was defined as the total number of complications that occurred in each cohort. The rate of postoperative blood transfusion refers to the rate of patients who received blood transfusion. We have previously described the criteria to remove abdominal drainage tubes^11^. The criteria to remove stomach drainage tubes included the following: (1) drainage volume less than 100 ml per day; (2) a lack of nausea and vomiting; and (3) after occurrence of first flatus. The discharge criteria were as follows: (1) the passing of at least 5 days since surgery; (2) successful administration of a semifluid diet and no need for intravenous nutrition; (3) a lack of complications or the presence of complications that did not require hospitalization; (4) the presence of sound mental status; and (5) the removal of all tubes.

**Table 2:** Operative outcomes of gastric cancer patients who underwent RADG or LADG.

Operative outcomes	RADG (n = 285)	LADG (n = 285)	*P* value
Total operative time, min	186 ± 12 (156–224)	147 ± 9 (126–168)	0.000^a^
Setup time, min	40 ± 3 (33–49)	12 ± 1 (10–17)	0.000^a^
Mini-invasive resection surgical time, min	106 ± 11 (77–136)	104 ± 8 (78–126)	0.276^a^
Laparotomy operation time, min	40 ± 3 (32–48)	30 ± 3 (25–41)	0.000^a^
Operative blood loss, mL	150 ± 151 (50–1000)	166 ± 139 (50–1300)	0.000^a^
Time to first flatus, hours	55.5 ± 6.5 (42.0–76.0)	56.4 ± 12.1 (39.0–80.0)	0.513^a^
Time to remove stomach tube, days	4.5 ± 4.1 (3.0–34.0)	5.0 ± 5.1 (3.0–35.0)	0.347^a^
Time to liquid diet, days	5.5 ± 4.1 (3.0–35.0)	6.0 ± 5.1 (3.0–36.0)	0.516^a^
Time to semi-liquid diet, days	6.6 ± 4.2 (3.0–36.0)	7.1 ± 5.2 (3.0–36.0)	0.606^a^
Postoperative volume of abdominal drainage, mL	397 ± 361 (150–4100)	520 ± 503 (200–3900)	0.000^a^
Time to remove abdominal drainage tube, days	6.3 ± 3.2 (4.0–35.0)	7.1 ± 4.5 (4.0–38.0)	0.002^a^
Perineural invasion, n (%)	116 (40.7)	114 (40.0)	0.864^a^
Lymphovascular invasion, n (%)	109 (38.2)	106 (37.2)	0.795^a^
Numbers of retrieved lymph nodes	26.4 ± 3.7 (18.0–34.0)	22.6 ± 3.8 (16.0–32.0)	0.000^b^
Postoperative blood transfusion, n (%)	6 (2.1%)	16 (5.6%)	0.030^c^
Postoperative length of stay, days	9.0 ± 4.5 (6.0–38.0)	9.5 ± 5.3 (5.0–40.0)	0.066^a^
Unplanned readmission, n (%)*	6 (2.1%)	7 (2.5%)	0.779^c^

*LADG*: laparoscopic assisted distal gastrectomy; *RADG*: robotic assisted distal gastrectomy.

*Within 30 days after operation.

^a^Mann–Whitney U test.

^b^Student’s t test.

^c^Pearson’s chi-squared test.

**Table 3:** Complications of gastric cancer patients who underwent RADG or LADG.

Complications	RADG (n = 285)	LADG (n = 285)	*P* value
Patients with complications, n (%)	30 (10.5%)	36 (12.6%)	0.432^a^
single complication	25 (8.8%)	26 (9.1%)	0.883^a^
multiple complications	5 (1.8%)	10 (3.5%)	0.191^a^
Overall complications, n (%)	35 (12.3%)	48 (16.8%)	0.123^a^
Surgical complications, n (%)	19 (6.7%)	36 (12.6%)	0.016^a^
wound infection/liquefaction	5 (1.8%)	6 (2.1%)	0.761^a^
delayed gastric emptying	3 (1.1%)	4 (1.4%)	1.000^b^
intestinal obstruction	0 (0.0%)	3 (1.1%)	0.247^b^
intra-abdominal hemorrhage	2 (0.7%)	2 (0.7%)	1.000^b^
intra-abdominal effusion/abscess	2 (0.7%)	2 (0.7%)	1.000^b^
duodenal stump leakage	5 (1.8%)	6 (2.1%)	0.761^a^
gastrojejunostomy anastomotic leakage	0 (0.0%)	3 (1.1%)	0.247^b^
gastrojejunostomy anastomotic bleeding	1 (0.4%)	1 (0.4%)	1.000^b^
gastroduodenal anastomotic bleeding	0 (0.0%)	1 (0.4%)	1.000^c^
pancreatic fistula	1 (0.4%)	8 (2.8%)	0.044^b^
General complications, n (%)	16 (5.6%)	12 (4.2%)	0.438^a^
deep vein thrombosis	2 (0.7%)	0 (0.0%)	0.479^b^
pulmonary embolism	1 (0.4%)	0 (0.0%)	1.000^c^
pneumonia	5 (1.8%)	6 (2.1%)	0.761^a^
pleural effusion	1 (0.4%)	3 (1.1%)	0.616^b^
heart failure	1 (0.4%)	1 (0.4%)	1.000^b^
myocardial infarction	1 (0.4%)	0 (0.0%)	1.000^c^
atrial fibrillation	1 (0.4%)	0 (0.0%)	1.000^c^
sepsis	1 (0.4%)	1 (0.4%)	1.000^b^
cerebral hemorrhage	1 (0.4%)	0 (0.0%)	1.000^c^
cerebral infarction	1 (0.4%)	0 (0.0%)	1.000^c^
urinary tract infection	1 (0.4%)	1 (0.4%)	1.000^b^
Clavien-Dindo classification, n (%)			
I	3 (1.1%)	7 (2.5%)	0.202^a^
II	16 (5.6%)	21 (7.4%)	0.395^a^
IIIa	8 (2.8%)	13 (4.6%)	0.266^a^
IIIb	4 (1.4%)	6 (2.1%)	0.523^a^
IV	1 (0.4%)	0 (0.0%)	1.000^c^
V	3 (1.1%)	2 (0.7%)	1.000^b^
≥III	15 (5.3%)	21 (7.4%)	0.302^a^
Reoperation, n (%)	5 (1.8%)	6 (2.1%)	0.761^a^
Mortality, n (%)	3 (1.1%)	2 (0.7%)	1.000^b^

*LADG*: laparoscopic assisted distal gastrectomy; *RADG*: robotic assisted distal gastrectomy.

^a^Pearson’s chi-squared test.

^b^Continuous correction chi-squared test.

^c^Fisher’s exact test.

### Surgical procedures

Distal gastrectomy with D2 lymph node dissection was performed by an extensively experienced team (TYL). The anesthesia method, patient’s position, and Trocar placement (“U” type) have been described previously^11^. All procedures were performed according to the Japanese Gastric Cancer Treatment Guidelines^19^. Ultrasonic shears were used in all robotic and laparoscopic surgeries. After *in vivo* dissociation, a 5–7-cm incision was made in the mid-upper abdomen. Surgeons chose anastomotic methods according to their experience and the residual stomach volume: (1) Billroth I digestive tract reconstruction was performed by gastroduodenostomy; one abdominal drainage tube was placed near the solid arrow. (2) Billroth II or Roux-en-Y gastrojejunostomy was performed, with suture and reinforcement of the duodenal stump performed in all cases. Two abdominal drainage tubes were placed near the duodenal stump and splenic recess. Finally, the abdominal wall was sutured layer by layer.

### Statistical analysis

EmpowerStats statistical software (http://www.empowerstats.com/en/) was used for PSM. The statistical analyses in this study were performed using SPSS 22.0 software (SPSS Inc, IBM, Armonk, NY, USA). Continuous variables were expressed as means ± standard deviation (SD) and range according to whether the variables obeyed normal distribution or not. A Student’s t-test or a Mann-Whitney U test was used to compare continuous variables between the RADG and LADG groups. A chi-squared test or Fisher’s exact test was used to compare categorical variables (numbers and percentages) between the two groups. *P* < 0.05 was considered statistically significant.

## Results

### Clinical baseline characteristics

Figure 1 shows a diagram of patient selection in the RADG and LADG groups. After PSM, 570 patients were enrolled in the current study, including 375 male and 195 female patients, with an average age of 57.07 years (range 41 to 80 years). Table 1 presents the clinical baseline characteristics of the included patients in the two groups. All characteristics (sex, age, body mass index (BMI), type of digestive tract reconstruction, depth of tumor invasion, pathological node stage, TNM stage, ASA classification, and diameter of the neoplasm) showed no significant differences between the RADG group and LADG group (*P* > 0.05).

### Short-term outcomes

The short-term outcomes of the patients in the two groups are displayed in Table 2. The total operative time was longer in the RADG group than in the LADG group (186 ± 12 (156–224) min vs. 147 ± 9 (126–168) min, *P* = 0.000). For the subgroup analysis based on operative time, the setup time and the laparotomy operation time were also longer in the RADG group than in the LADG group (*P* < 0.000), whereas the minimally invasive operation time was not significantly different between the two groups (106 ± 11 (77–136) min vs. 104 ± 8 (78–126) min, *P* = 0.276). The RADG group was associated with less blood loss during the operation than that of the LADG group (150 ± 151 (50–1000) vs. 166 ± 139 (50–1300) mL, P = 0.000), and the rates of postoperative blood transfusion were also reduced in the RADG group (2.1% vs. 5.6%, *P* = 0.030). The volume of postoperative abdominal drainage was significantly lower in the RADG group than in the LADG group (397 ± 361 (150–4100) vs. 520 ± 503 (200–3900) mL, *P* = 0.000). Compared with the LADG group, the time to removal of an abdominal drainage tube was also shorter in the RADG group (*P* = 0.008). Moreover, the number of lymph nodes retrieved from the RADG group (26.4 ± 3.7) was strikingly more than that from the LADG group (22.6 ± 3.8, *P* = 0.000). However, the time to first flatus, the time to removal of a stomach tube, the time to successful administration of a liquid or semi-liquid diet, the postoperative length of stay, and the occurrence of unplanned readmission within 30 days after surgery were not significantly different between the RADG group and LADG group (*P*å 0.05) (Table 2).

### Complications

Table 3 shows the postoperative complications and subgroups of complications of the patients in the two groups. After PSM, there were 66 patients (11.6%) with complications in the MIS cohort, 30 patients with complications in the RADG group and 36 patients in the LADG group, and the rates were comparable between the RADG and LADG groups (10.5% vs. 12.6%, *P* = 0.432). The overall rate of complications was not significantly different between the two groups (12.3% vs. 16.8%, *P* = 0.123). The rate of general complications was also similar in the two groups (*P* = 0.438). However, the rate of surgical complications was obviously lower in the RADG group (6.7% vs. 12.6%, *P* = 0.016). For analysis based on surgical complications subgroups, we found that the rate of postoperative pancreatic fistula was lower (*P* = 0.044), whereas the rates of other complications were similar in the RADG group and the LADG group. According to the update grading consensus of ISGPS^18^, one patient in LADG group and one patients in the RADG group were graded C POPF. And, other seven patients in the LADG group were graded B POPF. Regarding the severity (Clavien-Dindo classification) of complications, the results showed no difference between the two groups (*P*å 0.05). Furthermore, five patients in the RADG group and six in the LADG group underwent reoperation due to complications (*P* = 0.761). However, three patients died in the RADG group due to cerebral hemorrhage, extensive anterior myocardial infarction, and deep venous thrombosis with pulmonary embolism; two patients died in the LADG group owing to duodenal stump fistula with abdominal hemorrhage and pulmonary infection with heart failure. The mortality rates were similar between the two groups (*P* = 1.000).

## Discussion

Currently, reports in the field of robotic-assisted gastrectomy are increasing year by year, and most of them are observational studies with patients who presented at a relatively early stage of disease^20,21^. These studies commonly enroll patients with different TNM stages and who have underwent different types of stomach resection, and as such, there are many factors that may affect the authenticity of their results^22^. Another concern is that approximately half of the world’s gastric cancer cases occur in China, most of which are in an advanced stage (T2–4aN0-3M0) at the time of initial diagnosis, and China plays a major role in the global burden of gastric cancer^3,23,24^. Radical gastrectomy with D2 lymphadenectomy is the present standard surgical procedure for patients with local AGC in accordance with the Japanese Gastric Cancer Treatment Guidelines^19^. In the past two decades, a large number of studies including RCTs have shown that LG combined with D2 lymphadenectomy for AGC is safe and feasible and can achieve oncological outcomes that are similar to those of open surgery^5,7–9,25^. However, because they represent an emerging technology, we conducted a large sample retrospective cohort study to evaluate whether robots have advantages in the treatment of AGC. In this retrospective cohort study, we found that RADG resulted in less blood loss, less volume of abdominal drainage, less indwelling time of abdominal drainage tubes, and more harvested lymph nodes than LADG for AGC.

In the present study, we analyzed the total operation time and performed related subgroup analyses. The total operation time was significantly longer in the RADG group than in the LADG group, which was consistent with previous reports^26^. In further analyses of the operation time, we found that the setup time and the laparotomy operative time were longer in the RADG group, whereas the mini-invasive surgery resection time was similar between the two groups. This shows that the reason why the total operation time of the robot group is longer than that of non-robot-assisted groups lies in set up of the robot, and the real operative time is not significantly different between the two groups. The potential reasons for longer operative times were similar to those identified in a previous study^27^. Intraoperative blood loss is an important factor for assessing the quality of operation and indicates the rate of blood transfusion. Liu *et al*.^28^ reported similar outcomes to ours, with less blood loss in the robotic-assisted gastrectomy group, in their retrospective cohort study, and potential reasons for less blood loss have been described in previous publications^11^. Our study also found that the rate of blood transfusion was reduced in the RADG group compared to the LADG group. Several studies have indicated that perioperative blood transfusion is related to poor survival and more complications after operation^29,30^.

The recovery of gastrointestinal function is crucial for the recovery of patients after surgery^31^. The indicators representing the recovery of gastrointestinal function are mainly time to first flatus and successful administration of a liquid or semiliquid diet. However, the indexes of gastrointestinal function, such as the time to first flatus, were similar between the two groups in our present study. This result is mainly because the surgical operation area is mostly localized to the upper abdomen, which has less influence on the function of the bowel^11^. Both groups of patients underwent distal gastrectomy, and the same method was used for specimen removal and digestive tract anastomosis. This may be the underlying cause of the lack of a significant difference in stomach tube indwelling time between the two groups.

In addition, our study showed that the RADG group had less abdominal drainage and fewer days to removal of abdominal drainage tubes than the LADG group, which is consistent with our previous research^11^. In fact, almost no studies have compared the abdominal drainage of patients in the robotic and laparoscopic groups. With the advantage of the instrument, the robot can better maintain the anatomical level during surgery (in the mesogastrium space) and reduce the damage of the mesogastrium, thereby reducing the exudation of liquid^32^. D2 lymph node dissection is a difficult procedure in radical gastrectomy for AGC, particularly at the No. 7/8/9 stations. The lymph node count has been shown to be an indispensable factor for assessing the pathological stage correctly^33^. Another study showed that a higher lymph node count was related to better survival^34^. The present study identified that the number of harvested lymph nodes in the RADG group was statistically higher than that in the LADG group, which was less than in previous research^14^. The underlying cause for this discrepancy is that there is no fulltime pathologist to dissect lymph nodes from specimens in our hospital. However, the difference of retrieved lymph nodes between two groups is related to surgeon experiences, number of gastric cancer operations, learning curve and surgical technique^35^. In our center, gastrectomy with D2 lymph node dissection was performed by the same operation team with extensively experienced and the lymph node count was done by the same pathologist team. Therefore, our result about the differences in lymph nodes can provide some value for the clinic. However, the postoperative hospitalization time was shorter in the RADG group than that in the LADG group (9.0 ± 4.5 (6.0–38.0) vs. 9.5 ± 5.3 (5.0–40.0)), but the data without significantly difference (*P* = 0.066). Moreover, unplanned readmission within 30 days did not differ between the two groups (*P* > 0.05).

Another irreplaceable indicator for evaluating the safety, feasibility and quality of surgery is the rate of complications. In the present results, the rate of patient complications showed no remarkable difference between the two groups (10.5% vs. 12.6%, *P* = 0.432). This incidence of complications is similar to those identified in previous studies from South Korea^36^. The number of overall complications was also not significantly different between the RADG and LADG groups (12.3% vs. 16.8%, *P* = 0.123). This shows that in terms of total complications, the safety and feasibility of RADG are no less than those of LADG, although the robotic group included cases taken from the learning curve phase. Moreover, we further evaluated subgroups of complications. One amazing discovery is that the rate of surgical complications of the robotic group is obviously lower than that of the laparoscopic group (6.7% vs. 12.6%, *P* = 0.016). Interestingly, subsequent subgroup analyses of surgical complications revealed that the rate of pancreatic fistula was significantly lower in the RADG group than in the LADG group (0.4% vs. 2.8%, *P* = 0.044), which is consistent with previous studies^37,38^. The underlying cause for this difference may be the advantage that the robot has in its precision and stability, and it is not likely that the robot will damage the pancreas when cleaning lymph nodes and clearing the pancreatic capsule^22^. Ojima *et al*. indicated that amylase activity from abdominal drainage fluid were higher in the laparoscopic gastrectomy group than in the robotic group on postoperative day 1 (*P* = 0.028)^39^. However, patients with gastric cancer who do not have relevant clinical symptoms or who do not have partial pancreatectomy during the operation will not be routinely detected the level of amylase in the abdominal drainage fluid after operation in our center. There were five patients underwent reoperation in the RADG group, one patient underwent debridement and drainage operation due to POPF, one patient underwent duodetomy and drainage operation due to duodenal stump fistula, and three patients underwent suture hemostasis operation due to gastrojejunostomy anastomotic bleeding or intra-abdominal hemorrhage. And, there were six patients underwent reoperation in the LADG group, one patient underwent debridement and drainage operation due to POPF, one patients underwent suture hemostasis operation due to intra-abdominal hemorrhage, one patient underwent repair of duodenal stump and drainage operation, one patient underwent duodetomy and suture hemostasis due to duodenal stump fistula and intra-abdominal hemorrhage, and two patients underwent anastomotic repair of gastrojejunum and drainage operation due to gastrojejunostomy anastomotic leakage. In addition, we also analyzed the severity of complications between the two groups, and the results showed no difference. Unfortunately, there were 3 patients in the robot group, and 2 patients died in the laparoscopic group due to complications (*P* = 0.779).

Finally, we cannot deny the limitations of this study. First, although we used PSM, there may still be case selection bias because the study is a retrospective cohort study rather than an RCT. Second, because of insufficient follow-up time, we did not analyze the long-term oncology outcomes between the two groups. The above results need to be further confirmed by a multicenter RCT. Ojima *et al*. has registered a RCT of robotic vs. laparoscopic gastrectomy with lymphadenectomy for gastric cancer (number: UMIN000031536)^40^. Fortunately, our center has preregistered a multicenter RCT of robotic and laparoscopic distal gastrectomy with the Chinese Clinical Trial Registry (registration number: ChiCTR1900023933).

## Conclusions

In conclusion, the present research shows that RADG with D2 lymph node dissection for AGC is an alternative minimally invasive surgical procedure. Compared with LADG, RADG shows better short-term outcomes, including less operative blood loss, reduced rate of postoperative blood transfusion, less abdominal drainage, shorter time to removal of abdominal drainage tubes, and retrieval of more lymph nodes. The rates of surgical complications and pancreatic fistula were significantly lower in the RADG group than in the LADG group.

## Data Availability

Access to the database can be obtained from the corresponding author on reasonable request.
